# Supplementary material for: Cell type specific long non-coding RNA targets identified by integrative analysis of single-cell and bulk colorectal cancer transcriptomes
Source: Sci Rep. 2024 May 13;14:10939. doi: 10.1038/s41598-024-61430-7 (PMC11091208; doi:10.1038/s41598-024-61430-7)
Supplement: Supplementary file 1 — Supplementary Table 1. [file 41598_2024_61430_MOESM1_ESM.pdf]

**Suppl. Table 1**

| **CRISPRi sgRNA oligonucleotides** | | |
|---|---|---|
| Target | sgRNA | Sequence |
| AASV1 | sgRNA1 | GTCACCAATCCTGTCCCTAG |
| AASV1 | sgRNA2 | GTCCCCTCCACCCCACAGTG |
| CASC19 | sgRNA1 | AATGAATAACTTACCCAACC |
| CASC19 | sgRNA2 | AACTTACCCAACCTGGTAAG |
| LINC00460 | sgRNA1 | GGGGGACCGAGACCTATGAG |
| LINC00460 | sgRNA2 | GAGATGAGTCCCCCTGGCTG |
| **Expression oligonucleotides** | | |
| Target | Strand | Sequence |
| GAPDH | Forward | GCACCGTCAAGGCTGAGAAC |
| GAPDH | Reverse | TGGTGAAGACGCCAGTGGA |
| CASC19 | Forward | TTGGAGTGCCTGGGTTAGA |
| CASC19 | Reverse | CTGTCCTTGCCAGTGTCTT |
| LINC00460 | Forward | ATGCACACTTCTCGGCTAAG |
| LINC00460 | Reverse | GGTCGTAACCTTCGTTCTCATC |

**Suppl. Table 1.** Sequences of CRISPRi sgRNA oligonucleotides and expression oligonucleotides
